# Understanding the origin of stereoselectivity in the photochemical denitrogenation of 2,3-diazabicyclo[2.2.1]heptene and its derivatives with non-adiabatic molecular dynamics

**DOI:** 10.3762/bjoc.21.156

**Published:** 2025-10-06

**Authors:** Leticia A Gomes, Steven A Lopez

**Affiliations:** 1 Department of Chemistry and Chemical Biology, Northeastern University, Boston, Massachusetts, 02115, United States

**Keywords:** non-adiabatic molecular dynamics, photochemistry, quantum mechanical calculations, stereoselectivity

## Abstract

Photochemical denitrogenation reactions of bicyclic azoalkanes produce strained bicyclic compounds of interest to synthetic organic chemists. We report a computational study on the mechanism of diazabicyclo[2.2.1]heptenes to address long standing mechanistic questions. Indeed, the mechanism of these reactions has been disputed for over six decades. We employed non-adiabatic molecular dynamics (NAMD) simulations combined with state-of-the-art multireference quantum mechanical calculations to understand the photophysical properties and mechanisms of these diazabicyclo[2.2.1]heptenes. The energetically accessible lowest excitations are *n*_NN_(σ_CN_) → π* and range from 3.94–3.97 eV. From the >292 trajectories, the reaction proceeds through a dynamically concerted but asynchronous denitrogenation reaction. One σ_CN_ bond breaks along the S_1_ surface; the other σ_CN_ breaks after hopping to the S_0_. We identified two clusters of S₁/S₀ surface hopping points from these trajectories. In the first cluster, the methylene bridge is fully inverted relative to the reactant geometry. In the second cluster, the inversion is only partial, with one of the carbon atoms in the methylene bridge inverted relative to the reactant. We identified each cluster's corresponding minimum energy conical intersection (MECI), indicating at least two possible S_1_/S_0_-MECIs. Our dynamics simulations illustrate that inversion begins in the excited state immediately after the first σ_CN_ bond breaks. This inversion is driven by the atomic momenta acquired after the bond breaks. These dynamical effects promote the formation of the inverted housane, thereby explaining the observed selectivities. A minority of trajectories undergo thermal conversion in the ground state, producing the minor retained housane product from inverted housane/diradical.

## Introduction

Photochemical reactions utilize light as a sustainable energy source and are considered to be ‘green’ reactions [1–2]. Organic chromophores absorb light, accessing higher-lying excited state(s) that exhibit distinct reactivities, leading to bond breaking and formation, irreversibly producing energy-dense compounds [2–3]. One promising strategy to access strained compounds involves gas-evolution (e.g., CO or N_2_) because of the associated entropic driving force; this approach has had wide utility in photomedicine and organic synthesis. For example, photochemical decarboxylation has been employed to release carbon monoxide at relatively safer doses in biological systems using photoresponsive CO-releasing molecules (photo-CORMs) [4] because these have shown anti-inflammatory activity [5–6]. Photochemical denitrogenation of azoalkanes has been utilized in the stereoselective synthesis of strained compounds such as bicyclo[1.1.0]butane (BCB) and bicyclo[2.1.0]pentane (housane).

**Scheme 1:**
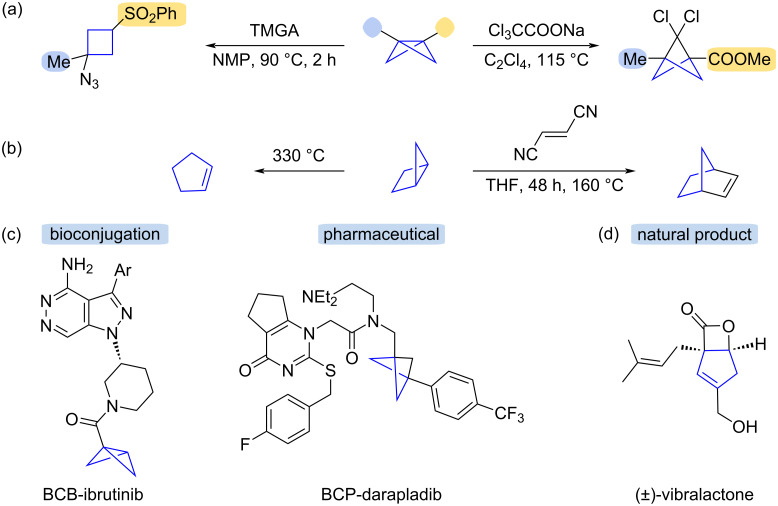
Applications of bicyclo[1.1.0]butane (a) and bicyclo[2.1.0]pentane (b). Molecules with biological activity are synthesized from bicyclo[1.1.0]butane (c) and bicyclo[2.1.0]pentane (d).

The energy stored in strained σ_CC_ bonds of BCB and housane makes them important building blocks in the synthesis of complex molecular structures. BCBs have been employed in ring-opening reactions with nucleophiles [7–11], radicals [12–15] and electrophiles [16–18] to give cyclobutanes and cyclobutenes [19–20], which are building blocks in regio- and stereoselective synthesis [21–27] (Scheme 1a). Additionally, BCB has been used in bioconjugation due to its high chemoselectivity for cysteine alkylation under mild conditions, for example, BCB-ibrutinib [28] (Scheme 1c). BCB is also used as a precursor to byciclo[1.1.1]pentanes, which are valuable motifs in drug design [19–20,29–33], such as BCP-darapladib (Scheme 1c) [30–31,34–35]. Housanes are versatile starting materials for different types of reactions including thermal isomerization to cyclopentenes [36–38] and 1,4-pentadienes [37–38], chemical electron-transfer oxidations to cyclopentene [39] and cycloaddition reactions with electron-deficient alkenes and alkynes [38,40–41] (Scheme 1b). Housane derivatives were also used in an atom-economical synthesis of the antibiotic [42–44] and potentially anti-obesity drug [45–49], (±)-vibralactone [42] (Scheme 1d).

**Scheme 2:**
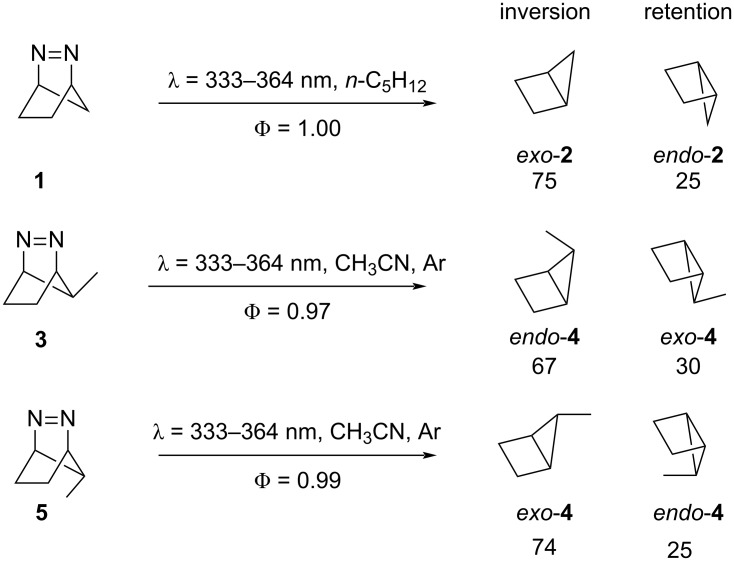
Diastereoselectivity in the direct photolysis of 2,3-diazabicyclo[2.2.1]hept-2-enes.

The thermolysis [50–60] and photolysis [57–58,60–78] mechanisms of cyclic azoalkenes have been studied with experimental and computational techniques for more than six decades, in solution [51,55,59–61,64,67,69–70,73–76,78–79], gas phase [50,55,62,64–65], and solid state [60,63]. The experimental studies on the thermal denitrogenation of diazabicyclo[2.1.1]hep-2-ene (DBH, **1**) indicate that both the parent compound and its derivatives undergo a concerted elimination of N_2_ and the inverted product (***exo*-2**) is preferentially formed [55–56]. Deuterium labeling of DBH was employed to experimentally measure the stereoselectivity of the reaction. In 1965, Crawford and co-workers experimentally found kinetic evidence for a 1,3-diradical from the thermal decomposition of **1** [56]. In 1963, Steel investigated the photolysis of diazabicyclo[2.1.1]hep-2-ene in solution, and the products were the same those obtained by thermolysis, nitrogen and bicyclo[2.1.0]pentane with quantum yield approaching unity [64]. Engel and co-workers explored further the reaction using transient spectroscopic methods and discovered that the rate of N_2_ formation is significantly slower than that the S_1_ fluorescence decay, suggesting that the N_2_ is not released at S_1_ state [80]. Roth and co-workers discovered a preference to form the inverted product ***exo*-2** from deuterated diazabicyclo[2.1.1]hex-2-ene (**1**) (Scheme 2) [71]. Adam and co-workers performed photochemical reactions with derivatives of **1**, including **3** and **5** (Scheme 2) through direct photolysis and with benzophenone as photosensitizer. No diastereomeric excess was observed [72] for reactions with photosensitizer. Trofimov and co-workers reported the temperature and solvent effect on the stereoselectivities [73–76] and observed a general preference for inversion across several derivatives of **1** [77–78]. These studies suggest that the thermal denitrogenation of diazabicyclo[2.1.1]hep-2-ene undergoes a concerted mechanism of elimination of nitrogen, while the photochemical reaction proceeds via a stepwise mechanism.

**Scheme 3:**
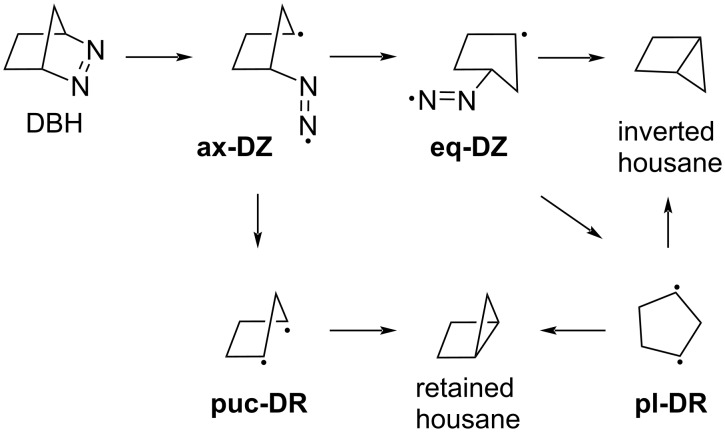
Mechanism for the photodenitrogenation of DBH proposed in the literature.

Quantum mechanical calculations have provided valuable insights into the photochemical stereoselectivities of cyclic azoalkenes. In 1998, Yamamoto and co-workers investigated the reaction paths for α-C–N and β-C–C bond cleavage during the direct and sensitized photolysis of DBH [81]. The minimum energy geometries in S_0_, S_1_, T_1_, and T_2_, conical intersections, transition structures, and singlet–triplet crossing were computed using CASSCF(10,8)/6-31G(d)//MP2/6-31G(d) [81]. The results suggested a stepwise C–N bond breaking with the formation of the diazenyl diradical intermediate [81]. In 2003, Olivucci and his co-workers investigated the inversion stereoselectivity of housane formation using CASPT2(12,10)/6-31G(d)//CASSCF(12,10)/6-31G(d). They also used a single classical trajectory with UB3LYP/6-31G(d) to probe the ground-state relaxation dynamics immediately after the conical intersection. They found that the axial diazenyl diradical (**ax-DZ** in Scheme 3) is selectively generated through a linear–axial conical intersection, providing access to five different reaction pathways. The computed energetics show a production of a cyclopentane-1,3-diyl diradical with a 1:1 production of retained and inverted housane, which disagrees with experimental observations. They argued that the DZ intermediate reacts before thermal equilibration. The formation of inverted housane occurs via the pseudo-axial-to-equatorial inversion of DZ. From the axial DZ, the puckered-DR (**puc-DR** in Scheme 3) radical could be formed resulting in retained housane. From the equatorial DZ (**eq-DZ** in Scheme 3), the inverted housane can be formed through a homolytic substitution (S_H_2) process or can be formed via a planar DR (**pl-DR** in Scheme 3) radical which affords retained and inverted housane [82].

In 2020, Rollins and co-workers also investigated the diradical pathways resulting from thermal decomposition using UM06-2X/6-31G(d,p) with quasiclassical trajectories and machine-learning analysis [83]. The results suggested a correlation between the out-of-plane bending of the methylene bridge and the stereoselectivity of the formation of retained and inverted housane [83]. Chen and Li explored the potential energy surfaces and surface crossing points of 2,3-diazabicyclo[2.1.1]hex-2-ene with CASPT2(12,10)/6-31G(d)//CASSCF(12,10)/6-31G(d,p) in 2006 and concluded that on the S_1_ surface one C–N bond is broken [84]. In 2011, Abe and co-workers investigated the denitrogenation mechanism of 7,7‐diethoxy‐2,3‐diazabicyclo[2.2.1]hept‐2‐ene, showing that a stepwise C–N-bond cleavage is energetically favored using broken‐symmetry (BS)‐(U)CCSD/6‐31G(d) and suggested that an equatorial conformation of the diazinyl diradical leads to the formation of the inverted product. An alternative route via an axial conformation of diazinyl diradical for the inverted product was suggested using Born–Oppenheimer molecular dynamics [85]. Most recently, our group performed multireference calculations with CASPT2(8,8)/ANO-S-VDZP//CASSCF(8,8)/ANO-S-VDZP and non-adiabatic molecular dynamics (NAMD) simulations of a series of diazabicyclo[2.1.1]hexenes. The minimum energy path showed stepwise σ_CN_ bond breaking and led directly to a minimum energy crossing point, corresponding to the inversion product. We also performed NAMD simulations on halogenated derivatives to test the role of hyperconjugative interactions; the NAMD simulations revealed that this stereoelectronic effect substantially perturbs the potential energy surface toward the retention products [86].

However, for 2,3-diazabicyclo[2.2.1]hept-2-enes, current theoretical reports only offer qualitative mechanistic understanding. The previous studies omit dynamical effects in the photochemical denitrogenation; the overall mechanism and origin of stereoselectivity are still unknown. We used state-of-the-art computations and NAMD simulations to understand the origin of the reactivities and stereoselectivities for a series of 2,3-diazabicyclo[2.2.1]hept-2-enes (Scheme 2). This report shows the first multiconfigurational NAMD simulation studies to understand the photochemical denitrogenation mechanism of 2,3-diazabicyclo[2.2.1]hept-2-enes.

## Results and Discussion

**Figure 1:**
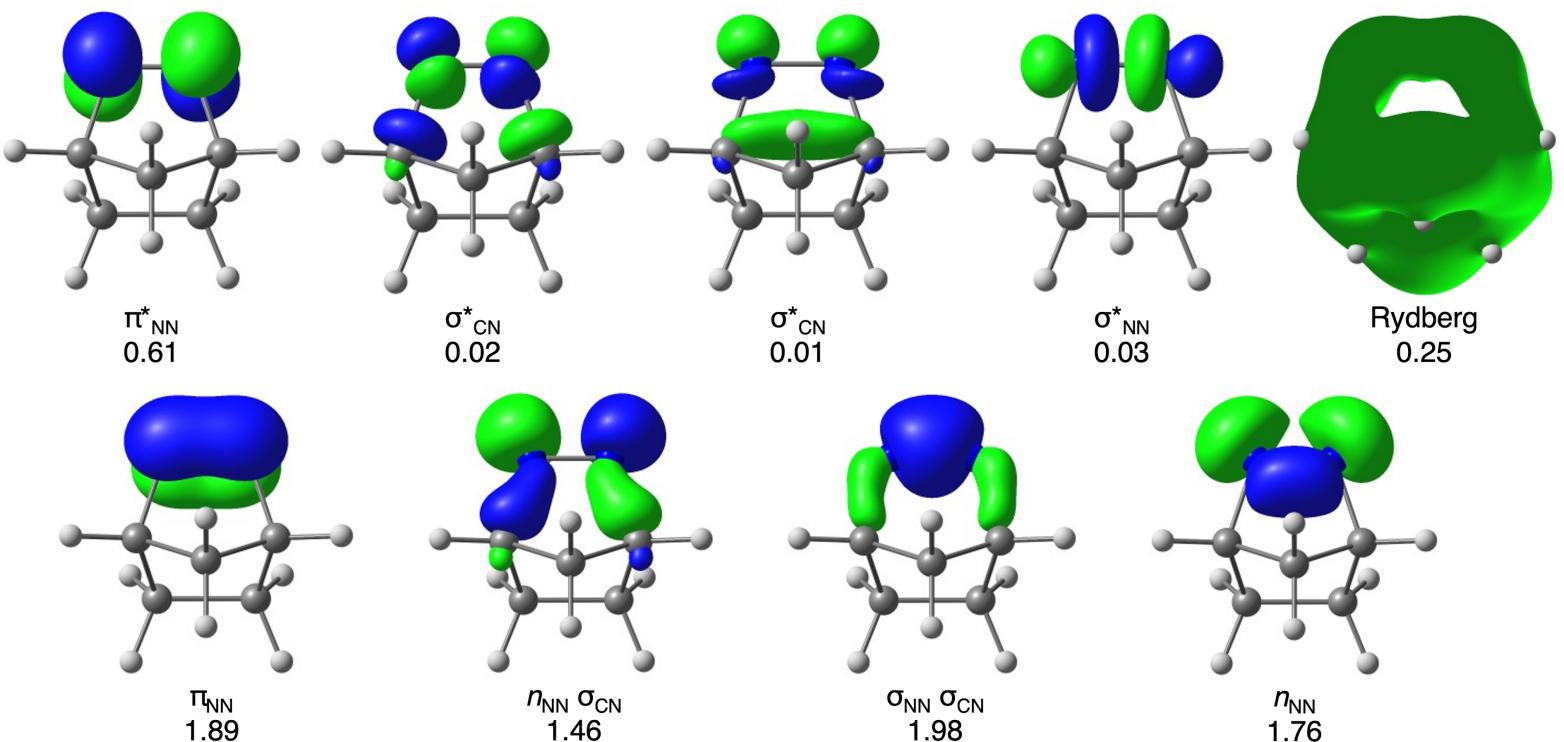
CASSCF(8,9) active space of **1** with average electron occupancies. Orbitals were calculated at the SA(4)-CASSCF(8,9)/ANO-S-VDZP level of theory. An isosurface value of 0.085 was used for all orbitals except Rydberg orbital, whose isosurface is 0.001.

We started our studies on characterizing the photophysical properties of **1**, **3**, and **5**. We considered the molecular orbitals and electrons most critical for describing the electronic structure of the molecules in the reaction. The CASSCF [87]/ANO-S-VDZP [88] active space for **1** is shown in Figure 1; we considered 8 electrons and 9 orbitals, along with their average occupancies. For derivatives **3** and **5** with a methyl substituent, the active space also consisted of 8 electrons and 9 orbitals, with their active space and occupancies available in Supporting Information File 1.

We benchmarked the vertical excitation energy with a (8,9)-active space against those obtained with time-dependent density functional theory by computing the vertical excitation energy, oscillator strength, and nature of the electronic transition of **1**, **3**, and **5** with ωB97X-D [89]/aug-cc-pVTZ [90], SA(4)-CASSCF(8,9)/ANO-VDZP, and SA(4)-XMS-CASPT2 [91] (8,9)/ANO-S-VDZP levels of theory, as shown in Table 1.

**Table 1:** Vertical excitation energies, oscillator strengths, and electronic transitions for the first three excited states of **1**, **3**, and **5** at TD-DFT, CASSCF, and XMS-CASPT2 levels of theory.

Molecule	Method	State	Energy (eV)	Wavelength (nm)	Oscillator strength	Nature

**1**	ωB97X-D/ aug-cc-pVTZ	S_1_	3.58	346	0.001	*n*_NN_(σ_CN_) → π*
		S_2_	6.23	199	0.001	*n*_NN_ → π*
		S_3_	6.34	195	0.013	*n*_NN_(σ_CN_) → Ryd

	XMS-CASPT2(8,9)/ ANO-S-VDZP	S_1_	3.97	312		*n*_NN_(σ_CN_) → π*
		S_2_	6.13	202		*n*_NN_ → π*
		S_3_	7.02	177		*n*_NN_(σ_CN_) → Ryd

	SA4-CASSCF(8,9)/ ANO-S-VDZP	S_1_	4.64	267	0.013	*n*_NN_(σ_CN_) → π*
		S_2_	6.95	179	0.000	*n*_NN_ → π*
		S_3_	7.96	156	0.039	*n*_NN_(σ_CN_) → Ryd

**3**	ωB97X-D/ aug-cc-pVTZ	S_1_	3.56	348	0.000	*n*_NN_(σ_CN_) → π*
		S_2_	6.13	202	0.000	*n*_NN_ → π*
		S_3_	6.38	194	0.008	*n*_NN_(σ_CN_) → Ryd

	XMS-CASPT2(8,9)/ ANO-S-VDZP	S_1_	3.94	315		*n*_NN_(σ_CN_) → π*
		S_2_	6.27	198		*n*_NN_ → π*
		S_3_	7.01	177		*n*_NN_(σ_CN_) → Ryd

	SA4-CASSCF(8,9)/ ANO-S-VDZP	S_1_	4.63	268	0.013	*n*_NN_(σ_CN_) → π*
		S_2_	6.92	179	0.000	*n*_NN_ → π*
		S_3_	8.02	155	0.033	*n*_NN_(σ_CN_) → Ryd

**5**	ωB97X-D/aug-cc-pVTZ	S_1_	3.55	349	0.001	*n*_NN_(σ_CN_) → π*
		S_2_	6.19	200	0.001	*n*_NN_ → π*
		S_3_	6.31	196	0.008	*n*_NN_(σ_CN_) → Ryd

	XMS-CASPT2(8,9)/ ANO-S-VDZP	S_1_	3.95	314		*n*_NN_(σ_CN_) → π*
		S_2_	6.42	193		*n*_NN_ → π*
		S_3_	7.04	176		*n*_NN_(σ_CN_) → Ryd

	SA4-CASSCF(8,9)/ ANO-S-VDZP	S_1_	4.63	268	0.013	*n*_NN_(σ_CN_) → π*
		S_2_	6.96	178	0.000	*n*_NN_ → π*
		S_3_	8.06	154	0.045	*n*_NN_(σ_CN_) → Ryd

The S_0_ → S_1_ vertical excitation energy, calculated using XMS-CASPT2(8,9)/ANO-S-VDZP, ranges from 3.94 to 3.97 eV and has the electronic transition from *n*_NN_(σ_CN_) to π*, with an oscillator strength of 0.013 for all molecules. The S_0_ → S_2_ excitation involves an *n*_NN_ → π* transition with all methods, and energy with XMS-CASPT2(8,9)/ANO-S-VDZP ranging from 6.13 to 6.42 eV with 0.000 oscillator strength. In contrast, S_0_ → S_3_ excitation has an *n*_NN_(σ_CN_) → Ryd electronic transition, with energy ranging from 7.01 to 7.04 eV with XMS-CASPT2(8,9)/ANO-S-VDZP and an oscillator strength ranging from 0.033 to 0.045. These results show agreement between the electronic nature of the transition of TD-DFT, CASSCF, and CASPT2, showing that the chosen active space can capture the photophysics of the molecules.

**Figure 2:**
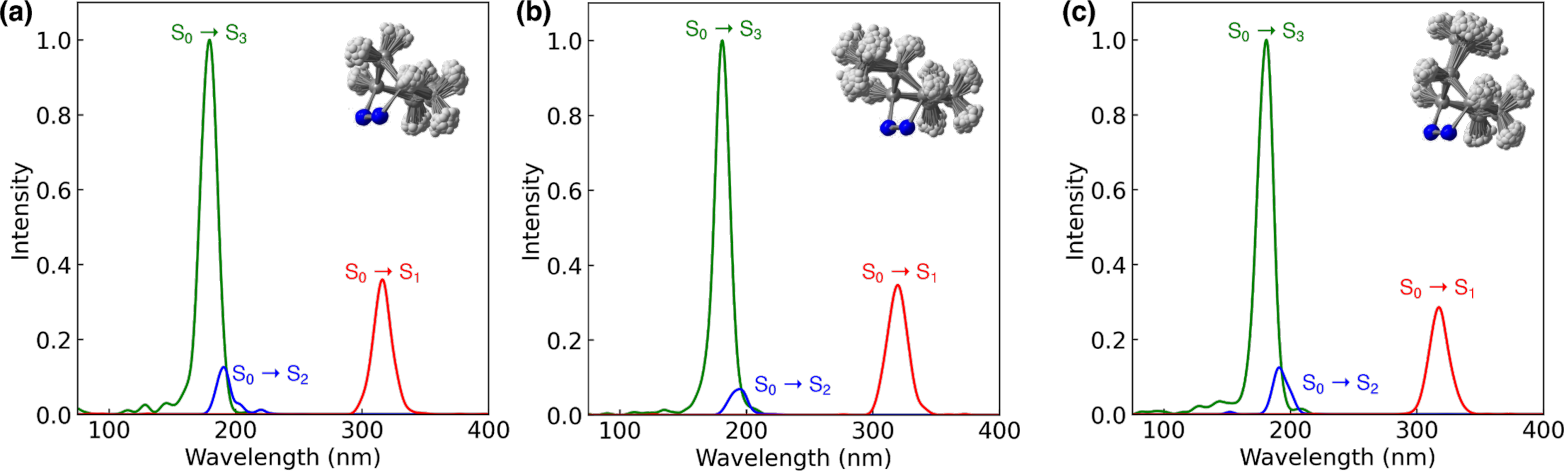
Absorption spectra and geometric overlays corresponding to Wigner-sampled geometries of **1** (a), **3** (b), and **5** (c) with XMS-CASPT2(8,9)/ANO-S-VDZP.

Table 1 provides the photophysical properties for the optimized ground-state geometry. However, experimentally, there is an ensemble of non-equilibrium geometries. To get a more realistic understanding of the absorption, we generated 500 geometries using the Wigner-sampling method to generate an ensemble of energetically accessible non-equilibrium geometries. We computed the gas-phase vertical excitation energies (S_0_ → S*_n_*, *n* = 1, 2, 3) and corresponding oscillator strengths of the Wigner-sampled structures of **1**, **3** and **5** to explore the nature of electronic excitations to the FC regions. This allowed us to predict an absorption spectrum with normalized oscillator strengths (S_0_ → S_3_). Figure 2 shows the computed absorption spectra of **1**, **3**, and **5**.

Figure 2 shows the **1**, **3**, and **5** computed absorption spectra with three λ_max_ peaks. The S_0_ to S_3_ has a peak centered from 179 to 181 nm and corresponds to a majority *n*_NN_(σ_CN_) → Ryd, the peak centered from 190 to 194 nm corresponds to a majority *n*_NN_ → π* from S_0_ to S_2_, and the peak centered from 316 to 319 nm corresponds to an *n*_NN_(σ_CN_) → π* from S_0_ to S_1_. The S_0_ to S_3_ has the highest intensity due to the allowed nature of the electronic transition n_NN_(σ_CN_) → Ryd. On the other hand, the S_0_ to S_1_ peak is expected to have a lower intensity because of the forbidden nature of the _NN_(σ_CN_) → π* electronic transition. This aligns with the trends observed in oscillator strength within the optimized geometry, where the S_0_ to S_3_ transition shows a higher oscillator strength than the S_0_ to S_1_ transition. In the case of the S_0_ to S_2_ transition, the spectrum reveals that a non-equilibrium assembled geometry produced a non-zero oscillator strength, unlike the optimized geometry, which exhibited zero. This difference is attributed to some mixing in the transition nature from S_0_ to S_2_, involving *n*_NN_(σ_CN_) → Ryd and *n*_NN_ → π* transitions. The S_0_ → S_1_ peaks from 316 to 319 nm are closer to the range of irradiation wavelengths of the experimentally used light source (333–364 nm). For **1**, this peak is closer to experimental absorbance, λ_max_ = 338 nm [80]. The remaining peaks, shown in blue and green for S_2_ and S_3_, respectively, absorb at higher energy than the experimental light source. Electronic transitions to these excited states are impossible in the experiment and thus will not be considered for this photochemical mechanistic study.

### DBH photodenitrogenation mechanism

**Figure 3:**
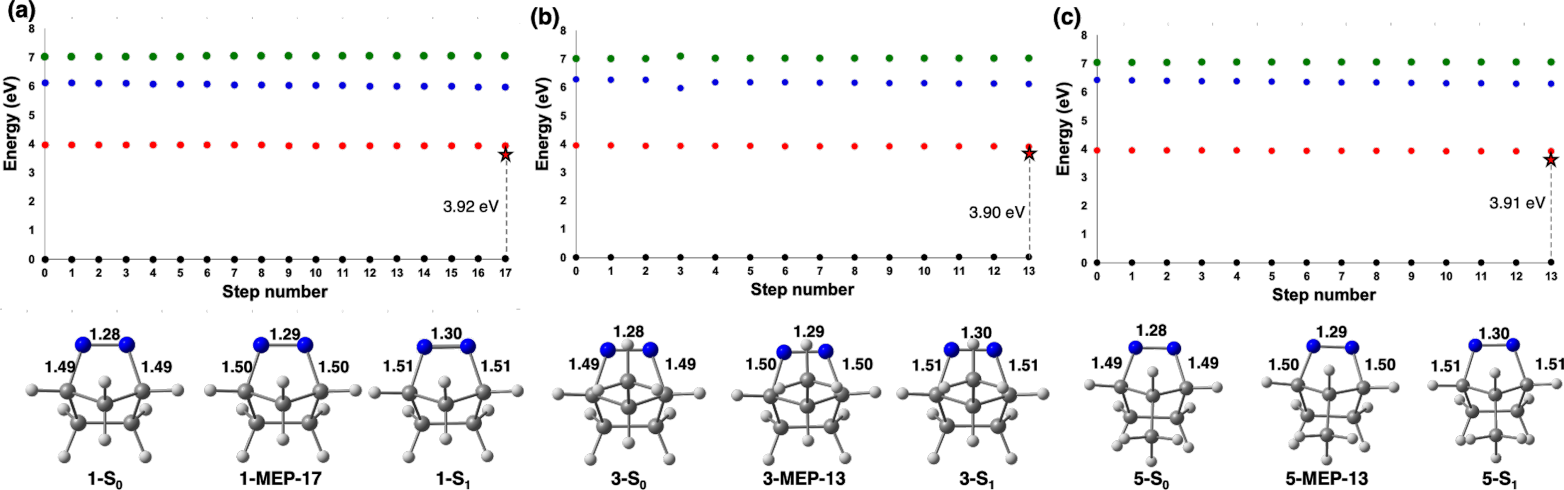
Minimum energy path using XMS-CASPT2(8,9)/ANO-S-VDZP for **1** (a), **3** (b), and **5** (c). The dots on the graphs correspond to the S_0_ (black), S_1_ (red), S_2_ (blue), and S_3_ (green) states. Relevant structures (S_0_ and S_1_ minima and endpoints of the MEP calculation) are shown below the plots. The C–N and N=N bond lengths are in angstroms on the structures shown. S_1_ energy is shown on the plot with a red star.

We then turned our attention to the photochemical reaction mechanisms leading to the formation of housane derivatives from 2,3-diazabicyclo[2.2.1]hept-2-enes. To determine the dominant mechanistic pathway, we started with a static exploration with the minimum steepest-descent energy path (MEP) from the FC-point along the S_1_ reaction coordinate. Figure 3 shows the MEP and the structure of S_0_, the last point of MEP, and S_1_ for **1**, **3**, and **5**. These structures are important for understanding the geometrical changes after light absorption and determining the dominant mechanism pathway, along the S_1_-reaction coordinate.

The MEP for **1** contains 17 geometries leading to the final structure **1-MEP-17**, and the MEP for **3** and **5** contain 13 geometries leading to final structures **3-MEP-13** and **5-MEP-13**, respectively. The dominant electronic transition is from *n*_NN_(σ_CN_) → π* for all molecules, and the geometrical changes along the S_1_-MEP correspond to an increased population of the π*_NN_ orbital. In all molecules, the π_NN_ bond length increased from 1.28 Å at the S_0_-optimized geometry to 1.29 Å at the last MEP structure, which is consistent with the electronic transition because the π_NN_* is being populated, resulting in a less stable π_NN_. The σ_CN_ increased by 0.01 Å along the S_1_-MEP for all molecules. The final MEP structure has a large S_1_–S_0_ energy gap ranging from 3.90 to 3.92 eV with XMS-CASPT2(8,9)/ANO-S-VDZP for all molecules. The large S_1_–S_0_ energy gap suggests that the dominant mechanistic pathway is toward an S_1_ minimum, as it does not indicate a possible crossing between the S_1_ and S_0_ potential energy surface. We used the last MEP structure as an input to optimize an S_1_ minimum. The S_1_ optimized geometries are shown in Figure 3 as **1-S_1_**, **3-S_1_,** and **5-S_1_**. Their energies are 3.83 to 3.87 eV above the S_0_ and have nearly identical structure and energy as the last point of the MEP. Based on this static MEP calculation, the experimental quantum yield (Φ = 0.97–1.00) is inconsistent with this static analysis, since it suggests a non-productive and fluorescence pathway. We hypothesize that these S_1_ minima are shallow excited-state minima that can be escaped towards a productive region of the conical intersection seam, or dynamical effects enable this reaction towards near-unity quantum yields. We performed non-adiabatic molecular dynamics (NAMD) simulations starting from the S_1_-FC region of **1**, **3**, and **5** to verify our hypotheses, identify the dynamical effects that enable this reaction, and enumerate all possible mechanistic pathways.

### Non-adiabatic molecular dynamics simulations

We performed an NAMD simulation using the Fewest Switches Surface Hopping (FSSH) algorithm [92–94], with OpenMolcas [95–96] and with Python Rapid Artificial Intelligence Ab Initio Molecular Dynamics (Pyrai^2^MD) [97–100] for **1**, **3**, and **5**. The NAMD simulation was performed with the CASSCF(8,9)/ANO-S-VDZP method to elucidate the origin of stereoselectivities and the overall singlet-mediated mechanism involved in denitrogenation. We generated initial conditions with an ensemble of 700 non-equilibrium geometries through Wigner sampling with 300 K velocities. The trajectories were then propagated from the S_1_ Franck–Condon region for 1 ps. We defined a complete trajectory as one that reached 1 ps with a maximum energy drift of 0.06 a.u., resulting in 352, 396, and 292 completed trajectories for molecules **1**, **3**, and **5**, respectively; 98–99% of these trajectories end in the S_0_.

**Figure 4:**
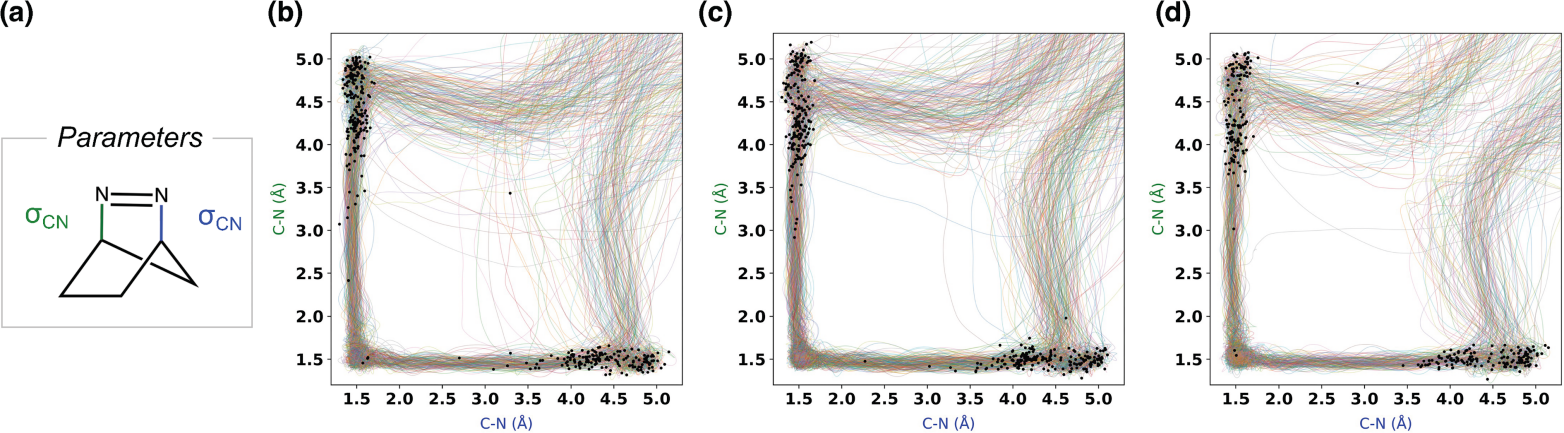
(a) The bond lengths we calculated are depicted. σ_CN_ bonds plotted against each other for **1** (b), **3** (c), and **5** (d). The solid multicolor lines on each plot show the bond lengths over time, and the dots represent the S_1_/S_0_ surface hopping points for each trajectory.

We wanted first to understand the synchronicity of the σ_CN_-bond breaking and whether bond-breaking occurs on the S_1_ and/or S_0_ state(s). To address these questions, we plotted the two σ_CN_ bond lengths throughout the trajectories (Figure 4). We observed that one σ_CN_ bond breaks first, as indicated by an increase in the σ_CN_ bond length. For the majority of the trajectories, when the broken σ_CN_ bond length reaches 4.5 to 5.0 Å, we observed the breaking of the second bond, suggesting a sequential bond-breaking process. This observation aligns with previous computational studies on the photodenitrogenation of DBH. The black dots represent the hopping points, the geometries where the trajectory crosses from S_1_ to S_0_. Figure 4 shows that the first σ_CN_ bond breaks on the S_1_ surface, while the second σ_CN_ bond breaks after relaxation to S_0_. We determine that the denitrogenation is stepwise but on an ultrafast timescale, making it dynamically concerted. By analyzing the state population of the trajectories over time, the obtained time constants were 174 fs for **1**, 163 fs for **3,** and 201 fs for **5**. The plots are available in Supporting Information File 1.

**Figure 5:**
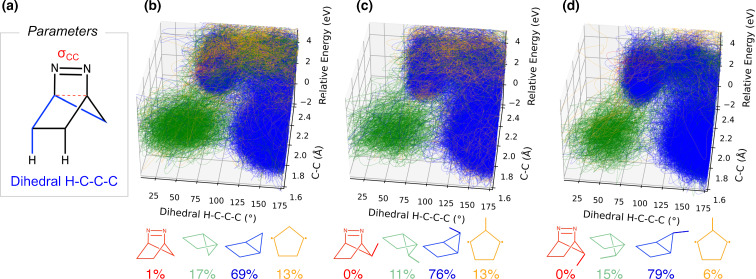
(a) Geometrical parameters. Plots show trajectories for a 1 ps NAMD simulation with CASSCF (8,9)/ANO-S-VDZP, monitoring C–C-bond formation, H–C–C–C dihedral angle, and relative energy for **1** (b), **3** (c), and **5** (d). The energy is relative to the lowest total energy of each trajectory. The red trajectories return to reactant, the green trajectories form retained housane, the blue trajectories form inverted housane, and the yellow trajectories lead to a diradical intermediate. The quantum yield for each species is provided below its structure.

Next, we aimed to elucidate the reaction pathways originating from the S_1_ Franck–Condon region for **1**, **3**, and **5**. We classified the final geometries of each trajectory to identify the resulting products and computed their respective quantum yields (QYs). We identified four pathways following the S_1_ to S_0_ crossing: the reversal to reactant, the inversion product, the retention product, and a diradical intermediate, as shown in Figure 5. Each pathway is shown in a different color, with the products shown at the bottom in their corresponding colors along with the quantum yields. Our computations indicate high photodenitrogenation quantum yields, ranging from 0.98 to 0.99, which align closely with the experimental QYs observed between 0.97 and 1.00. Some trajectories terminated prematurely at a diradical intermediate. We attempted to extend these trajectories by an additional 0.5 ps; however, most encountered convergence failures or exhibited energy drift exceeding 0.06 a.u. In the one case where the trajectory successfully reached 1.5 ps with conserved energy, we observed the formation of both inverted and retained housane, consistent with the existence of an intermediate. Nevertheless, due to the challenges in consistently achieving 1.5 ps extensions while maintaining energy conservation, these trajectories were excluded from the statistical analysis of the housane quantum yield.

The inverted housane exhibited the highest quantum yields, ranging from 0.69 to 0.79, consistent with the experimentally observed preferred product. Notably, all trajectories leading to retained housane passed through a region characterized by dihedral angles exceeding 110°, corresponding to the inverted housane if the C–C bond is formed and to the inverted diradical if it is not. This observation offers new insights into the reaction mechanism, showing that inverted housane/diradical forms initially in the ground state and can thermally convert into retained housane. This finding represents a significant shift in mechanistic understanding, highlighting the importance of incorporating dynamical effects. Previous studies, summarized in Scheme 3, predicted that a diazenyl diradical (**az-DZ**) with one broken C–N bond would retain the stereochemistry of the reactant, leading directly to the formation of retained housane. However, this pathway was not observed when dynamical effects were included in our simulations. Our predicted inverted-to-retained housane ratios – 4, 7, and 5 for **1**, **3**, and **5**, respectively – are higher than the experimental ratios of 3, 2, and 3. We attribute this overestimation to the limited simulation time of 1 ps. Since retained housane forms via thermal conversion from inverted housane/diradical, longer timescales are likely required to capture its formation accurately. Nevertheless, we maintained the 1 ps simulation length to ensure energy conservation, as energy drift increases with longer trajectory times, and to obtain a statistically representative number of trajectories.

**Figure 6:**
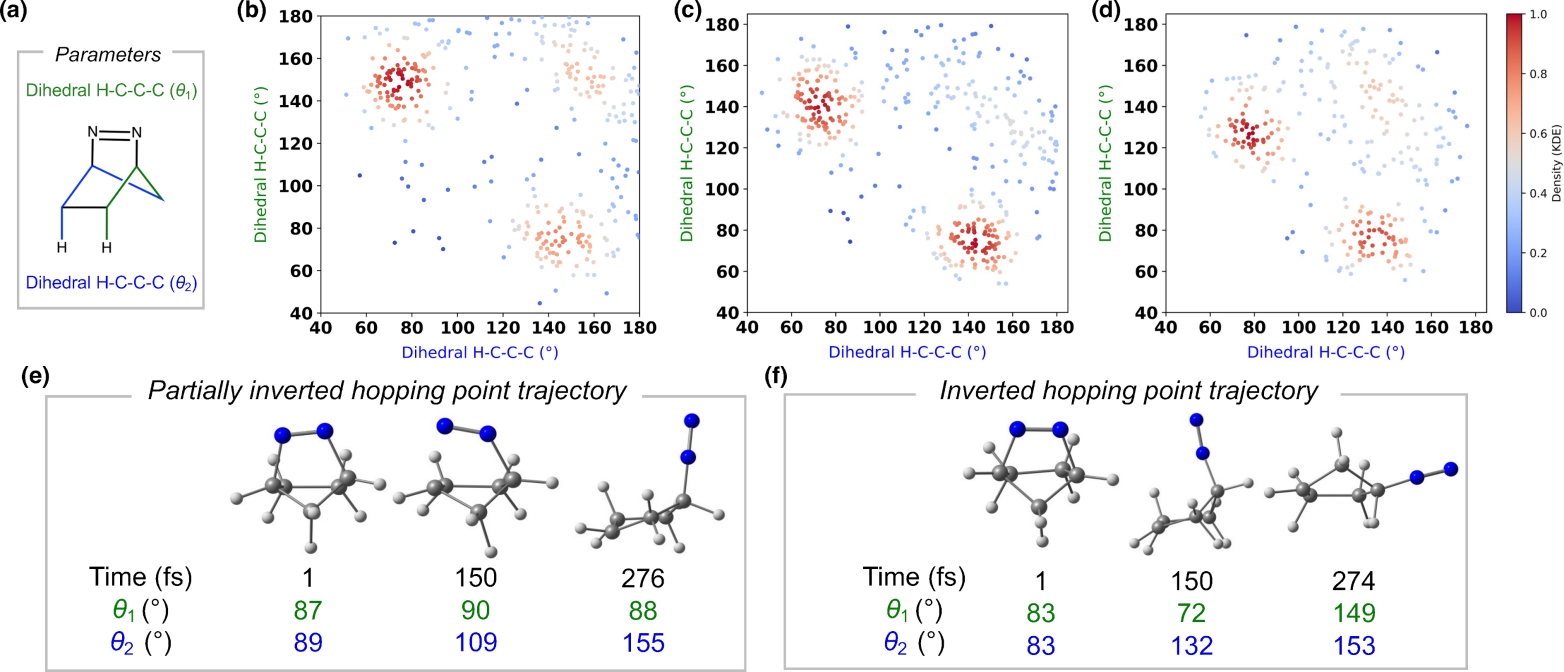
(a) Geometrical parameters. H–C–C–C dihedral angles plotted against each other for S_1_-to-S_0_ hopping point geometry of **1** (b), **3** (c), and **5** (d). Representative trajectories for partially inverted hopping point (e) and completely inverted hopping point (f).

We analyzed the S_1_/S_0_ crossing by examining the geometries of the hopping points to understand the stereoselectivities of the inverted housanes. We plotted the two H–C–C–C dihedral angles, shown in Figure 6a, and estimated the local density for each hopping point using kernel density estimation, as shown in Figure 6b–d. By plotting the dihedral angles, we identified two types of hopping points: one that is partially inverted and another that is fully inverted. The H–C–C–C dihedral angles of the reactant-optimized geometry range from 81° to 83° for compounds **1**, **3**, and **5**. By plotting the dihedral, two types of hopping points were identified. The first type, termed partially inverted hopping point, features one dihedral angle similar to that of the reactant, ranging from 60° to 90° across all molecules. In contrast, the other dihedral angle is significantly larger, with the highest density localized between 140° and 160°, 130° and 150°, and 120° and 140° for compounds **1**, **3**, and **5**, respectively. The lower dihedral angle ranges observed for the largest dihedral angle for derivatives **3** and **5** suggest that the methyl substituent on the methylene group restricts dihedral inversion. This restriction may arise from increased steric hindrance imposed by the methyl group on the methylene bridge of the derivatives. Two clusters represent the partially inverted hopping point, depending on which σ_CN_ breaks first. The second type, referred to as the inverted hopping point, occurs when both dihedral angles are significantly larger than those of the reactant, with both dihedral angles ranging from 140° and 160°, 130° and 150°, and 120° and 140° for compounds **1**, **3**, and **5**.

We selected representative trajectories from **1** to illustrate different types of hopping points. Figure 6e shows snapshots from a trajectory that leads to a partially inverted hopping point. At 1 fs, both dihedral angles are similar, with θ₁ at 87° and θ₂ at 89°. By 150 fs, the first σ_CN_ bond breaks. The dihedral angle θ₂, corresponding to the carbon still bonded to N₂, increases to 109°, while θ₁ rises slightly to 90°. At the hopping point (276 fs), θ₁ remains nearly unchanged at 88°, but θ₂ increases significantly to 155°, indicating that the carbon bonded to N₂ is already inverted and positioned above the molecular plane. In contrast, the other carbons are in the plane or below it; we consider this hopping point geometry partially inverted. A trajectory snapshot leading to a fully inverted hopping point is shown in Figure 6f. At 1 fs, both dihedral angles are identical at 83°. At 150 fs, the σ_CN_ bond is broken, θ₁ decreases slightly to 72°; θ₂ increases substantially to 132°, placing the carbon bonded to N₂ above the molecule plane. At the hopping point (274 fs), both θ₁ and θ₂ are significantly increased (149° and 153°, respectively). Here, all carbons of the methylene bridge are located above the molecular plane, indicating complete inversion of stereochemistry.

**Figure 7:**
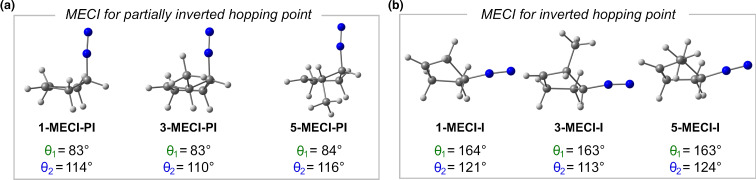
The minimum energy conical intersection geometries are shown for the partially inverted hopping point (a) and the inverted hopping point (b), along with their dihedrals for **1**, **3**, and **5**.

Using kernel density estimation [101], we found that partially inverted hopping points can be found in high-density regions, plotted in red within their respective clusters across all molecules. The observed geometric differences and distinct clustering patterns suggest the existence of two separate S₁ → S₀ crossing channels. To test this hypothesis and investigate the preference for partially inverted hopping geometries, we performed optimizations of the minimum energy conical intersection (MECI). The MECI for the partially inverted hopping point (**MECI-PI**) and the inverted hopping point (**MECI-I**) for **1**, **3**, and **5** are shown in Figure 7.

The **MECI-PI** exhibits θ₁ between 83° and 84°, and θ_2_, a more elongated dihedral angle, ranging from 110° to 116°. In contrast, **MECI-I** displays two elongated dihedral angles: θ₁ ranges from 163° to 164°, and θ_2_ from 113° to 124°. The significant geometrical difference supports the hypothesis of two crossing regions between the S_1_ and S_0_. The **MECI-PI** has lower energy for all molecules studied than the **MECI-I**, by being lower in energy by 0.15 eV, 0.19 eV, and 0.09 eV for **1**, **3**, and **5**, respectively. These results suggest that the gradient towards the **MECI-PI** is steeper than for **MECI-I**, making the pathway to the **MECI-PI** more favorable. All MECI structures show partial stereochemical inversion. Following the initial σ_CN_-bond cleavage, the carbon atom still bonded to N₂ begins to move towards inversion, indicating that dynamic effects help promote the stereoselective inversion.

## Conclusion

We used multiconfigurational quantum mechanical calculations and NAMD simulations to investigate the mechanism of photodenitrogenation of 2,3-diazabicyclo[2.2.1]heptene and its derivatives, which produce the strained compound housane, important building blocks in the synthesis of complex molecular structures. We identified that the key electronic transitions are S_0_ → S_1_ (*n*_NN_(σ_CN_) → π*) transitions, with excitation energies ranging from 3.94 to 3.97 eV. MEP calculations indicate that the dominant mechanistic pathway leads to an S_1_ minimum, which is inconsistent with the nearly unity quantum yield observed experimentally. We employed NAMD to explore all possible mechanistic pathways and identify the dynamic effects that enable this reaction. The predicted quantum yields ranged from 0.98 to 0.99, aligning closely with the experimentally observed quantum yields of 0.97 to 1.00. Our simulations reveal that all productive trajectories involve a dynamically concerted but asynchronous denitrogenation reaction, characterized by breaking one σ_CN_ bond in the S_1_ state and the second σ_CN_ bond in the S_0_ state. After the first σ_CN_ bond breaks, the stereochemistry of the carbon bonded to N_2_ begins to invert, suggesting that dynamic effects promote this inversion. The diazenyl diradical, which shares the same stereochemistry as the reactant – and was proposed in prior studies – was not observed when dynamic effects were included in our simulations. Following the S_1_/S_0_ surface hopping points, we identified four distinct pathways: reversion to the reactant, formation of the inverted housane, formation of the retained housane, and formation of the diradical, which presumably undergoes ring-closing if given sufficient simulation time. These were unfortunately prohibitively expensive due to the CASSCF computation of the energy, gradients, and non-adiabatic couplings at each 0.5 fs timestep. Our results indicate that the retained stereochemistry of housane is generated in the ground state via thermal conversion from inverted housane/diradical.

## Computational Details

### Single reference methods

We used density functional theory (DFT) to optimize the ground-state global minima of **1**, **3**, and **5**. The molecular geometries were optimized using B3LYP/6-31(G) [102–103]. Time-dependent density functional theory (TD-DFT) was used to calculate the vertical excitation energies, wavelengths, and oscillator strength for the first 10 excited singlet states of **1**, **3**, and **5**. The TD-DFT calculations were performed using the range-separated hybrid density functional ωB97X-D [89] with the aug-cc-pVTZ [90,104] basis set. All single reference methods were run using the Gaussian 16 software [105].

### Multiconfigurational methods

The multiconfigurational calculations were performed with a state-average complete active space self-consistent field (SA-CASSCF) [106–107] using OpenMolcas 19.11 [95–96]. The methods are described with the format SA(N)-CASSCF(*m*,*n*), where N is the number of the singlet states the calculation is averaged over. The variables *m* and *n* denote the number of electrons and orbitals used in the active space, respectively. For all included molecules, we used an (8,9) active space consisting of one π and π* orbitals, one *n*_NN_ σ_CN_, one σ_NN_ σ_CN_, two σ*_CN_, one σ*_NN,_ one *n*_NN,_ and a Rydberg orbital. The active space is shown in Figure 1, and Figures S_1_ and S_2_ in Supporting Information File 1 for **1**, **3**, and **5**, respectively. We employed SA(4)-CASSCF(8,9)/ANO-S-VDZP [88] multiconfigurational calculations to optimize the geometries of both the ground and excited states, determine vertical excitation energies, oscillator strengths, and nature of the electronic transitions. Additionally, we conducted minimum energy path (MEP) calculations to track the reaction pathway along the excited state starting from the Franck–Condon (FC) point. All optimized structures were verified as stationary points by calculating their vibrational frequencies, ensuring no imaginary frequencies were present. To include partial dynamic correlation, we performed single-point energy calculations using extended multistate complete active space perturbation theory (XM2-CASPT2) [91] on the CASSCF-optimized geometries.

### Non-adiabatic molecular dynamics (NAMD)

We performed NAMD using the fewest switches surface hopping (FSSH) [92–93] to investigate the photochemical pathways of compounds **1**, **3**, and **5** along the potential energy surfaces (PESs) of both ground and three excited states. The initial nuclear positions and velocities were sampled based on the Wigner distribution [108] at 300 K. All quantum mechanical calculations are performed in OpenMolcas using SA(4)-CASSCF(8,9)/ANO-S-VDZP. With the Python Rapid Artificial Intelligence Ab Initio Molecular Dynamics (PyRAI^2^MD) [97–99], we used FSSH to run the NAMD simulations. Trajectories were initiated from the S_1_ Franck–Condon (FC) region, with a total simulation duration of 1 picosecond and a time step of 0.5 femtoseconds.

## Supporting Information

File 1 Details for the active space for derivatives, time constants, the outcome of extended trajectories ending at the diradical intermediate, and the energy of minimum energy conical intersections (MECIs).

## Data Availability

All data that supports the findings of this study is available in the published article and/or the supporting information of this article. All optimized structures, output files for vertical excitation energy, MEP and dynamics (including energies, xyz, velocities for each trajectory) are available at https://doi.org/10.6084/m9.figshare.25326076
