# Supplementary material for: Understanding the origin of stereoselectivity in the photochemical denitrogenation of 2,3-diazabicyclo[2.2.1]heptene and its derivatives with non-adiabatic molecular dynamics
Source: Beilstein J Org Chem. 2025 Oct 6;21:2007–20. doi: 10.3762/bjoc.21.156 (PMC12522161; doi:10.3762/bjoc.21.156)
Supplement: File 1 — Details for the active space for derivatives, time constants, the outcome of extended trajectories ending at the diradical intermediate, and the energy of minimum energy conical intersections (MECIs). [file Beilstein_J_Org_Chem-21-2007-s001.pdf]

BEILSTEIN JOURNAL OF ORGANIC CHEMISTRY

Supporting Information

for

Understanding the origin of stereoselectivity in the photochemical denitrogenation of 2,3-diazabicyclo[2.2.1]heptene and its derivatives with non-adiabatic molecular dynamics

Leticia A. Gomes and Steven A. Lopez

Beilstein J. Org. Chem. **2025,** *21,* 2007–2020. doi:10.3762/bjoc.21.156

Details for the active space for derivatives, time constants, the outcome of extended trajectories ending at the diradical intermediate, and the energy of minimum energy conical intersections (MECIs)

License and Terms: This is a supporting information file under the terms of the Creative Commons Attribution License (https://creativecommons.org/licenses/by/4.0). Please note that the reuse, redistribution and reproduction in particular requires that the author(s) and source are credited and that individual graphics may be subject to special legal provisions.
The license is subject to the *Beilstein Journal of Organic Chemistry* terms and conditions: (https://www.beilstein-journals.org/bjoc/terms)

Table of contents

Active spaces...S1

Dynamics results..S2

Active spaces

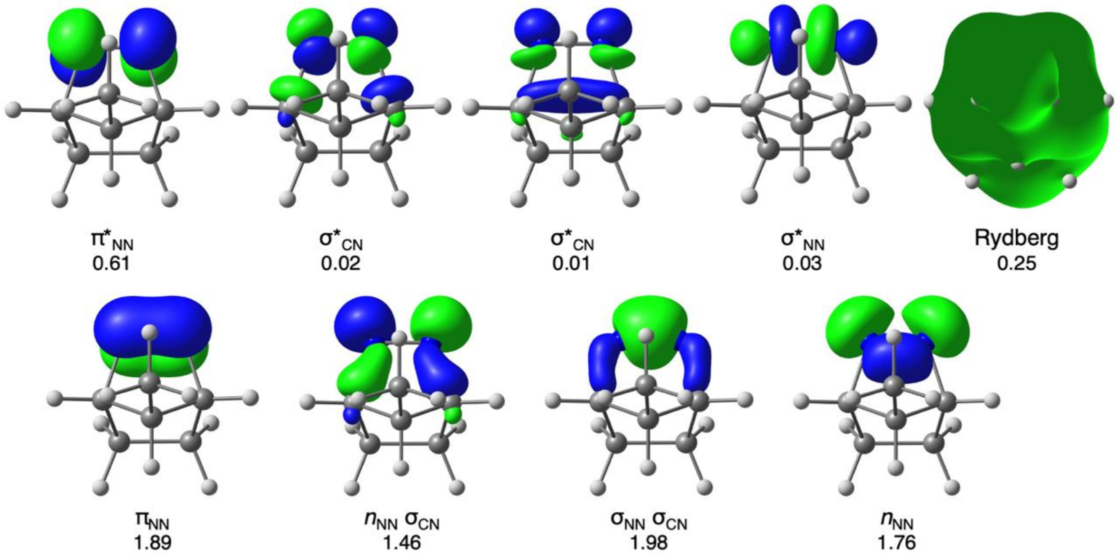

Figure S1: CASSCF(8,9) active space of **3** with average electron occupancies. Orbitals were calculated at the SA(4)-CASSCF(8,9)/ANO-S-VDZP level of theory. An isosurface value of 0.085 was used for all orbitals except Rydberg orbital, whose isosurface is 0.001.

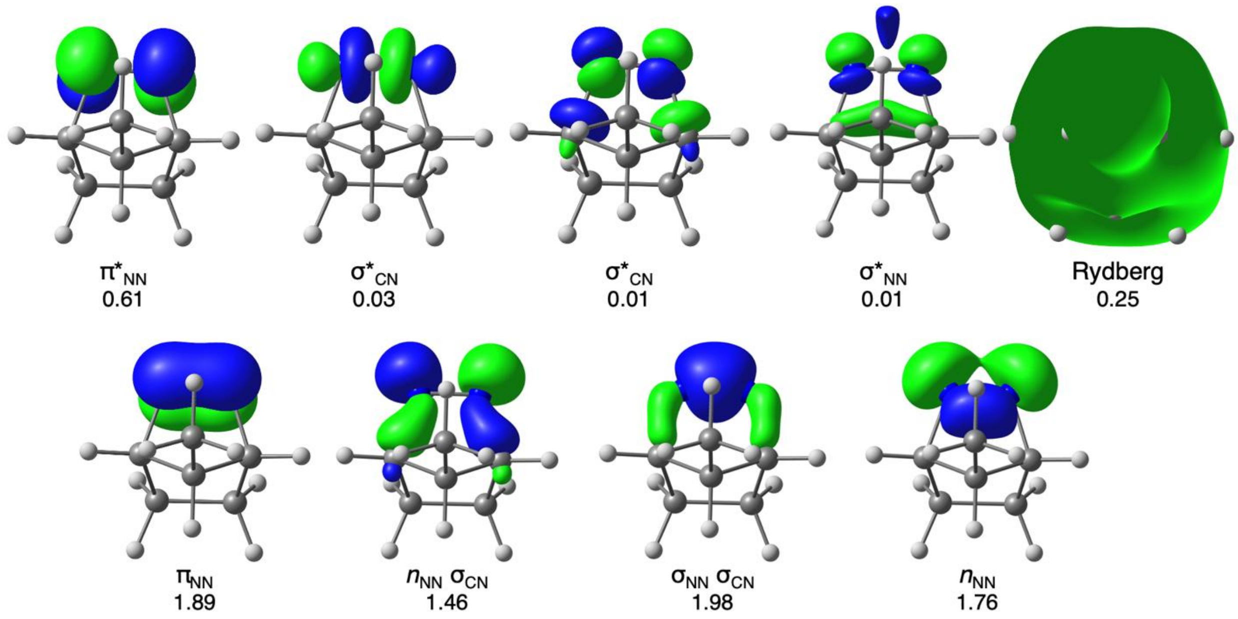

Figure S2: CASSCF(8,9) active space of **5** with average electron occupancies. Orbitals were calculated at the SA(4)-CASSCF(8,9)/ANO-S-VDZP level of theory. An isosurface value of 0.085 was used for all orbitals except Rydberg orbital, whose isosurface is 0.001.

Dynamics results

Table S1: Number of trajectories with 2000 fs that ended in each state for **1**, **3**, and **5** obtained with SA(4)-CASSCF(8,9)/ANO-S-VDZP.

Molecule	S_0	S_1	S_2	S_3	Total number of trajectories
1	350	2	0	0	352
3	390	6	0	0	396
5	289	3	0	0	292

The classification of the final product follows a hierarchical decision-making framework based on geometrical parameters, systematically filtering structures through progressively more specific criteria. The process begins by identifying reactants, defined as structures where both σ_{CN} values are less than 2.0, indicating no bond breaking. If this criterion is not met, the next classification step distinguishes 1-bond species, where only one of the σ_{CN} values is less than 2.0, suggesting that the reaction is incomplete for trajectories ending on this geometry. Structures that do not fall into either of these categories are then evaluated for a diradical classification, which is assigned when the σ_{CC} distance exceeds 2.0, indicating that no C–C bond has formed. In contrast, both σ_{CN} bonds have been broken. For structures that do not exhibit these bonding characteristics, the classification proceeds based on stereochemical parameters: if the H–C–C–C dihedral angle is less than 110.0 degrees, the product is categorized as retained housane, meaning it preserves its original stereochemistry. Finally, if none of the preceding conditions apply and the dihedral angle is equal to or greater than 110.0 degrees, the structure is classified as inverted housane, indicating a stereochemical rearrangement.

Table S2: Number of trajectories with 2000 fs that ended in each species for S_0 of **1**, **3** and **5** obtained with SA(4)-CASSCF(8,9)/ANO-S-VDZP.

Molecule	Inverted Housane	Retained Housane	Ratio Inverted/Retained	Diradical	Reactant
1	241	61	3.95	45	3
3	299	42	7.11	49	0
5	228	43	5.30	17	1

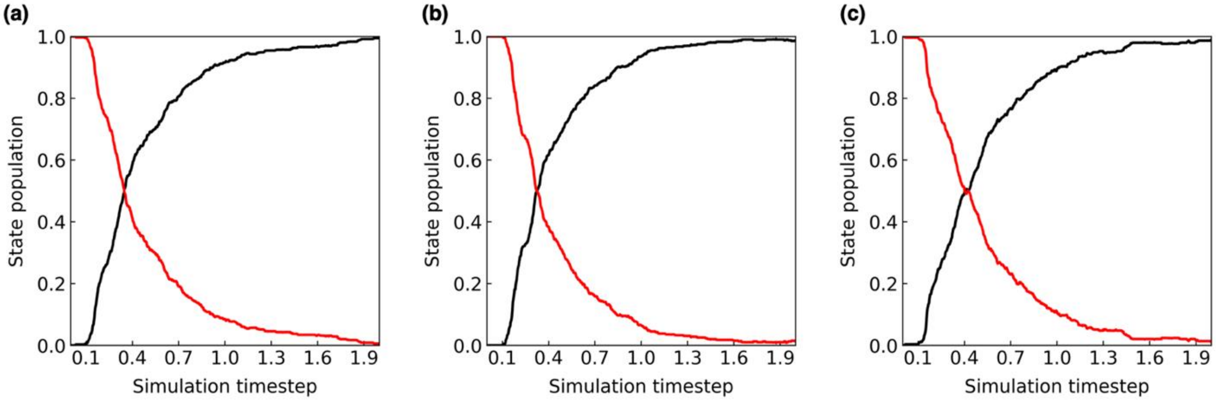

Figure S3: Population distribution for **1**, **3** and **5** obtained with SA(4)-CASSCF(8,9)/ANO-S-VDZP.

The half-life is 174 fs for **1** (a), 163 for **3** (b) and 201 for **5**(c).

Table S3: Number of trajectories with 1.5 ps for trajectories that ended initially on **DR** that ended in each species for S_0 of **1**, **3** and **5** obtained with SA(4)-CASSCF(8,9)/ANO-S-VDZP.

Molecule	Inverted Housane	Retained Housane	Diradical	Reactant	Convergence issue	Not conserved
1	8	9	14	0	6	8
3	21	5	8	0	15	4
5	2	2	5	0	4	4

Table S4: Energies in Hartree for each type of minimum energy conical intersection obtained with SA(4)-CASSCF(8,9)/ANO-S-VDZP for **1**, **3** and **5**.

Molecule	Energy in Hartree	
	MECI-PI	MECI-I
1	-302.8904162	-302.8849577
3	-341.9334338	-341.9265152
5	-341.9318822	-341.9287440
